# Retinoic Acid Receptor-Related Orphan Receptors: Critical Roles in Tumorigenesis

**DOI:** 10.3389/fimmu.2018.01187

**Published:** 2018-05-31

**Authors:** Jinshuo Fan, Zhilei Lv, Guanghai Yang, Ting ting Liao, Juanjuan Xu, Feng Wu, Qi Huang, Mengfei Guo, Guorong Hu, Mei Zhou, Limin Duan, Shuqing Liu, Yang Jin

**Affiliations:** ^1^Key Laboratory of Respiratory Diseases of the Ministry of Health, Department of Respiratory and Critical Care Medicine, Union Hospital, Tongji Medical College, Huazhong University of Science and Technology, Wuhan, China; ^2^Department of Thoracic Surgery, Union Hospital, Tongji Medical College, Huazhong University of Science and Technology, Wuhan, China

**Keywords:** retinoic acid receptor-related orphan receptors, RORα, RORβ, RORγ, cancer

## Abstract

Retinoic acid receptor-related orphan receptors (RORs) include RORα (NR1F1), RORβ (NR1F2), and RORγ (NR1F3). These receptors are reported to activate transcription through ligand-dependent interactions with co-regulators and are involved in the development of secondary lymphoid tissues, autoimmune diseases, inflammatory diseases, the circadian rhythm, and metabolism homeostasis. Researches on RORs contributing to cancer-related processes have been growing, and they provide evidence that RORs are likely to be considered as potential therapeutic targets in many cancers. RORα has been identified as a potential therapeutic target for breast cancer and has been investigated in melanoma, colorectal colon cancer, and gastric cancer. RORβ is mainly expressed in the central nervous system, but it has also been studied in pharyngeal cancer, uterine leiomyosarcoma, and colorectal cancer, in addition to neuroblastoma, and recent studies suggest that RORγ is involved in various cancers, including lymphoma, melanoma, and lung cancer. Some studies found RORγ to be upregulated in cancer tissues compared with normal tissues, while others indicated the opposite results. With respect to the mechanisms of RORs in cancer, previous studies on the regulatory mechanisms of RORs in cancer were mostly focused on immune cells and cytokines, but lately there have been investigations concentrating on RORs themselves. Thus, this review summarizes reports on the regulation of RORs in cancer and highlights potential therapeutic targets in cancer.

## Introduction

Cancer incidence and mortality rates are increasing worldwide with the growing and aging of the population, as well as risk factors such as outdoor pollution, tobacco smoke, and physical inactivity (1). Due to early detection and advanced treatments, cancer survival rates continue to grow, although a better understanding of carcinogenesis may lead to more effective treatment options for cancer.

The nuclear receptors (NRs) have been demonstrated to play essential roles in cancer-related progresses and to be potential therapeutic targets for many malignancies (2–5). The retinoic acid receptor-related orphan receptors (RORs) are a subfamily of the thyroid hormone receptor, which is a subfamily of the NRs and belonging to the orphan NR family (6). The ROR subfamily contains three members: RORα (NR1F1), RORβ (NR1F2), and RORγ (NR1F3).

Members of the RORs are typically regarded as noteworthy in inflammation, autoimmune diseases, metabolism disorders, circadian rhythms, development of neuron cells, and immune cell differentiation. Although RORs share some common sequences, the three RORs present a wide assortment of features. RORα and RORγ are important regulators of the immune system. For instance, the development and differentiation of Th17 cells are dependent on these factors (7–9). Moreover, studies show that RORγ is expressed in lymphoid tissue inducer cells, innate lymphoid cells, invariant natural killer T cells, and γδ T cells, which contribute to inflammation and autoimmune disease (10).

RORα, RORβ, and RORγ are all involved in the modulation of circadian rhythms. RORα functions as a positive regulator of the circadian modulator Bmal1 through binding to ROR-responsive elements (ROREs) (11, 12). RORβ mRNA expression levels were found to oscillate with true circadian rhythms, peaking at night-time (13), and modulation of circadian rhythms was disrupted in RORβ-deficient mice (14). Recent studies have proposed that RORγ1, but not RORα, is periodically expressed, and RORγ regulates several clock genes, such as Cry1, Bmal1, and Npas2, directly in a Zeitgeber time-dependent manner through these ROREs (15, 16).

Accumulating evidence shows that RORα and RORγ are involved in lipid/glucose metabolism, insulin sensitivity, and cardiometabolic control (17). A report showed that RORα could repress the transcriptional activity of PPARγ, leading to dysregulation of hepatic lipid metabolism (18). Recently, studies have shown that metabolic disorders affected by circadian rhythms might be attributed to RORα and RORγ, partly because of their modulation in both circadian and metabolic diseases. Moreover, earlier studies suggested that RORα was directly involved in melatonin-mediated anti-fibrotic processes (19) and beneficial manipulation in diabetic cardiomyopathy (20).

The expression sites and producing cells of RORs are also distinct from each other, consistent with their functions in the various diseases mentioned above. RORα and RORγ are expressed in all skin cell types, including epidermal keratinocytes, melanocytes, dermal fibroblasts, and several established lines of malignant melanomas. The expression levels of RORα/γ are dependent on the skin cell type and can be regulated by hydroxy derivatives of vitamin D3 (5, 21–24). Vitamin D3 formation is regulated by UVB (25); vitamin D3 metabolites are inverse agonists for RORα/γ; therefore, RORα and RORγ expression level could be regulated by UVB (5).

Other expression sites of RORα include the liver, skin, pancreas, brain, adipose tissue, islet cells, and the pineal gland. In addition to its expression and modulation in melanoma described above, RORα has been researched in breast cancer (BC) (26), melanoma (5), hepatocellular carcinoma (HCC) (27), and colon cancer (28). RORβ is mainly expressed in the brain and pineal gland (29). RORβ is upregulated or downregulated in cancers such as primary leiomyosarcoma of the uterus (30), a pharyngeal cancer cell line (31), and colorectal cancer (28). RORγ is expressed in the thymus and lymphoid organs, and RORγ production in cancer cells is detected in lung cancer (4), lymphoma (32), melanoma (5), and BC (33).

The RORs have been widely investigated in cancer and have shown varying influences in cancer-related processes, these differences may be due to their structures and their tissue-specific expression. Some studies suggest that RORα is a tumor suppressor and a potential therapeutic target for BC; and based on the limited researches on RORβ in cancer, RORβ might be a tumor suppressor as well. Others have proposed that activating RORγ may exert antitumor immunity (34), while RORγ is considered as protumor candidates in prostate cancer and lung cancer (4, 35). In this review, we summarize and discuss the structures of RORs and their roles in cancer-related processes, highlighting the potential therapeutic targets for cancer treatment.

## Structure and Ligands of RORs

The three ROR family members contain sequences similar to the retinoic acid receptor, with certain differences. The three ROR family members contain sequences similar to the retinoic acid receptor, but in minor details, the structures of each are distinct (36). The RORα gene maps to human chromosome 15q22.2, covering a large genomic region of 730 kb and generating four human RORα isoforms: RORα1—RORα4, while only RORα1 and RORα4 are found in mice (17). The RORβ and RORγ genes map to human q21.13 and 1q21.3, covering 188 and 24 kb, respectively. RORβ and RORγ each generate two isoforms: RORβ1/RORβ2 and RORγ1/RORγ2 (RORC2 in human and RORγt in mice). The isoforms of RORs differ in their amino terminals due to alternative exon splicing and promoter usage and their distinct expression and function in different tissues. However, if cells co-express RORs, the co-expressed RORs may overlap in several functions.

Receptor-related orphan receptor genes encode proteins of similar amino sequences ranging from 459 to 556 amino acids according to the different isoforms, and they all consist of four domains. These domains include an N-terminal domain, a highly conserved DNA-binding domain, a ligand-binding domain (LBD), and a hinge between the domains. Transcription is regulated by binding to RORE as a monomer (36).

No cognate ligands of RORs had been identified until crystallography studies on the LBD of RORα indicated that cholesterol and cholesterol sulfate function as natural ligands (37). Several retinoids, including all-trans retinoic acid and the synthetic retinoid ALRT 1550 (ALRT), have been identified to bind RORβ, reversibly and with high affinity (38). Thus, the retinoids have been identified as ligands of RORβ, although their specific regulation is not clearly understood. RORγ has been found to be co-expressed with RORα, and the ligands of RORα and RORγ have been reported as sterols or their derivatives and secosteroids (5, 6). Endogenously produced novel D3 hydroxy derivatives can act as both “biased” agonists of the vitamin D receptor and inverse agonists of RORα/γ (22), and hydroxylumisterols can act as ligands of RORα and RORγ (39). Melatonin was once considered a ligand for RORα (40, 41). However, contrasting reports showed that melatonin was not a natural ligand for RORα because melatonin could not activate RORα directly (42, 43). The docking scores calculated from molecular modeling of interactions between melatonin and its metabolites with RORα and RORγ predicted weak binding affinities (5), and the structures of melatonin and its metabolites were not similar to the sterols that were identified as natural ligands (37).

Except for the natural ligands of RORs mentioned above, there are also some synthetic RORγ ligands with therapeutic potential identified in literatures (6, 44). For instance, the inverse agonists of RORα and RORγ, SR2211 has been reported to inhibit the expression of IL-17A and cell viability in lung cancer (4) and suppress inflammation in a collagen-induced arthritis mouse model (45). And RORα and RORγ agonist SR1078 can induce cancer cell apoptosis and p53 stability (46). Inverse agonists or agonists like these two are promising therapeutic reagents for the diseases that RORs involved in, but there are still lack of studies to investigate their treatment potentials in cancer.

## Cancer Relevance

As illustrated above, RORs have been implicated in autoimmune or immune-mediated disease, the circadian rhythm, and metabolic disorders. RORs are also important regulators in various cancers due to their pivotal roles in immunity, the circadian rhythm, and metabolic homeostasis, which contribute to tumor progression.

**Figure 1:**
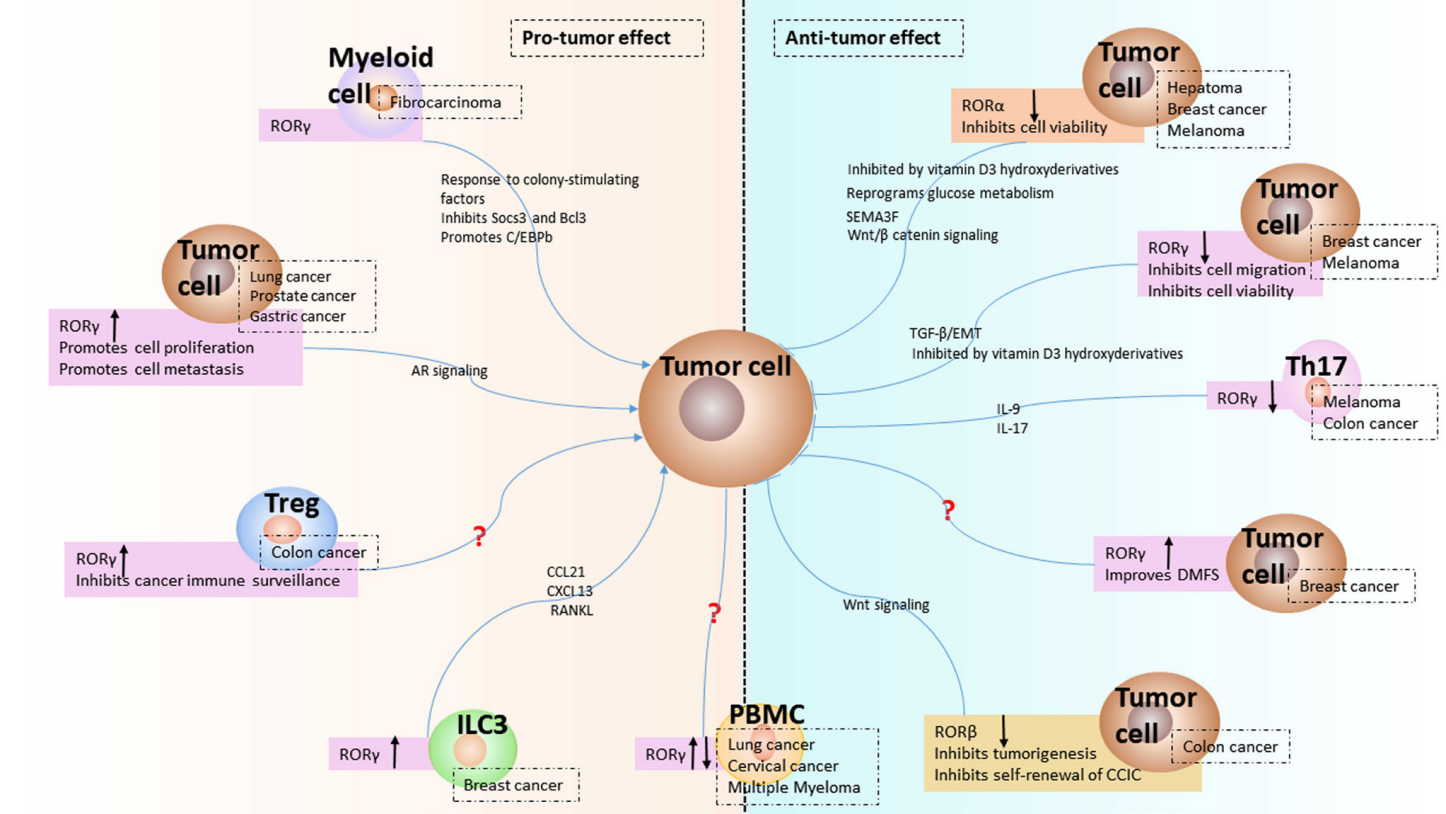
Expression and function of receptor-related orphan receptors (RORs) in tumor microenvironment. The expression of RORα and RORβ from tumor cell and the modulated expression of RORγ in group 3 innate lymphoid cells (ILC3), Th17, regulatory T cell (Treg), myeloid cell, and tumor cell from tumor microenvironment are presented as reviewed in the text. The downregulation of RORα and RORβ induce antitumor effect in hepatoma, breast cancer (BC), melanoma, and colon cancer. The upregulation of RORγ in ILC3 leads to protumor effect by chemokines in BC. The downregulation of RORγ in Th17 indicates antitumor effect by IL-17 in colon cancer. The upregulation of RORγ in Treg shows protumor effect in colon cancer. The expression of RORγ in myeloid cell has protumor effects *via* Socs3, Bcl3, and C/EBPb. The expression of RORγ in tumor cell is either increased or decreased depending on the cancer type. Increased expression of RORγ in lung cancer, prostate cancer, and gastric cancer results in protumor effect, while decreased expression of RORγ in BC and melanoma could induce antitumor effect *via* TGFβ/epithelial–mesenchymal transition (EMT) or vitamin D3 derivatives. The question mark refers to unknown mechanisms. The up or down black arrow refers to upregulation or downregulation. Antitumor: inhibits tumor progression; protumor: promotes tumor progression.

RORα has been found to be downregulated in keratinocyte-derived skin cancer (47) and is expressed in prostate cancer cells (48), melanoma cell lines (5, 49), and BC (50) (Table 1). Decreased expression of RORα is positively related with melanoma progression and shorter disease-free and overall survival (23, 24). RORα is also involved in inhibiting cell proliferation as a tumor suppressor (51). In human hepatoma cells, RORα was found to be upregulated after hypoxia induction (52), while RORα expression was lower in tumor tissues than in adjacent tumor tissues. It was also determined to be involved in the reprogramming of glucose metabolism and inhibiting hepatoma growth both *in vitro* and in a xenograft model *in vivo* (53). However, in one report, the production of RORα mRNA in colorectal cancer patients was unchanged (54), while RORα phosphorylation was found reduced and might be involved in colon cancer progression (55). In another report about BC, RORα was found to be downregulated, and low expression of RORα mRNA was associated with a poor prognosis (26). RORα is commonly considered a repressor (Figure 1), according to investigations into its role in cancer illustrated above.

**Table 1:** Studies of RORs in cancer.

Isoforms	Cancer type	Study population/model	Expressing cell	Expression level and biologic effects	Reference
**RORα**					

RORα	BC	BC tissues	BC cell	Activates aromatase expression Promotes cell proliferation in ER-positive BC	(50)

RORα	BC	Malignant and nonmalignant breast tissues	BC cell	Decreased Correlated with poor prognosis Inhibits cell invasion and regulates SEMA3F	(26)

RORα	Hepatoma	HCC and adjacent non-tumor tissue	Hepatoma cell	Decreased Reprograms glucose metabolism; inhibits hepatoma growth both *in vitro* and in a xenograft model *in vivo*	(53)

RORα	Colorectal cancer	Human colorectal tumors	Colorectal cancer cell	Unchanged	(54)

RORα	Colon cancer	Human colon tumors	Colon cancer cell	Attenuates Wnt/β-catenin signaling	(55)

RORα1	Prostate cancer	Prostate cancer cell line	Prostate cancer cell	Activation of RORα1 reduces 5-LOX expression might interfere with the mitogenic activity of fatty acids on prostate cancer	(48)

RORα4	Skin cancer	SSCC tissues	SCC cell	Decreased	(47)

RORα4	Melanoma	Human melanoma cell lines	Melanoma cell	Expressed in WM-98, WM-164, and SCBE2 cells	(49)

RORα	Melanoma	Human melanoma cell lines	Melanoma cell	As receptors for 20-hydroxy- and 20,23-dihydroxyvitamin D	(5)

RORα	Melanoma	Melanoma tissues	Unspecified	Decreased Positive associated with melanoma progression and shorter disease-free and overall survival	(24)

RORα	Melanoma	Benign (nevi) and malignant (melanomas) melanocytic tumor tissues	Keratinocytes, melanoma cells	Decreased Higher nuclear levels of RORα correlated with significantly longer overall and disease-free survival time	(68)

RORα4	Hepatoma	Hepatoma cell line	Hepatoma cell	Upregulated by hypoxia in HepG2 cells	(52)

**RORβ**					

RORβ	Colorectal cancer	Human primary colorectal cancer tissues	Colorectal cancer cell	Decreased Attenuate self-renewal of CCICs by binding with HBP1 promoter regions Enhance the HBP1-dependent inhibition of TCF4-mediated transcription and Wnt activity	(29)

RORβ	Colorectal cancer	Human colon cancer cell clones	Human colon cancer cell clones	Decreased	(28)

RORβ	Neuroblastoma	Neuronal cell line	Neuroblastoma cell	Binds to ROREs with low affinity Instigates transcription efficiently in Neuro2A but not in HeLa nuclear extracts due to an extract specific factor in Neuro2A	(56)

RORβ	Uterine leiomyosarcoma	Primary and metastatic uterine leiomyosarcoma tissues	Unspecified	Increased in primary tumor than metastatic tumor	(30)

RORβ	Pharyngeal cancer	Pharyngeal cancer cell line	Metastatic (Detroit 562) pharynx carcinoma cell	Increased Regulated by TLR3	(31)

**RORγ**					

RORγ	Lymphoma	RORγ^−/−^, RORγ^+/−^, and wild-type mice		Deficiency of RORγ leads to T cell lymphoma, metastasis, and death	(57)

RORγ	Multiple myeloma	PB and BM of patients with multiple myeloma	Lymphocytes	Unchanged	(58, 59)

RORγ	Multiple myeloma	Patients with multiple myeloma tissues	PBMC	Increased	(60)

RORγ	BC	Human BC tissues	Unspecified	Overexpressed among IL-17^Hi^ tumors	(61)

RORγ	BC	Human IDC tumor tissues	Tumor-infiltrating CD4^+^ and CD8^+^ T lymphocytes	Increased RORC and IL-17A expression is correlated in breast tumor tissues	(62)

RORγ	BC	BC tissues	ILC3	Increased Correlated with LN metastasis	(63)

RORγ1	BC	BC patients and cell line	BC cell	Positively associated with DMFS rate	(33)

RORγ	BC	TCGA and GEO BC collection, BC cell lines	BC cell	Decreased Negatively regulates the oncogenic TGF-β/EMT and mammary stem cell (MaSC) pathways and positively regulates DNA-repair Higher RORγt expression displayed increased probability of RFS	(64)

RORγ	BC	BC cell lines MAINZ data sets and UNC metastatic BC data set	BC cell	Increased Inversely correlated with PRMT2 expression Increased expression improved DMFS	(33)

RORγ	Melanoma	B16F10 mouse melanoma model	T cell	High IL-9 expression in RORγ^−^ T cells leads to inhibition of melanoma	(67)

RORγ	Melanoma	Human invasive melanomas tissues, skin samples (neonatal and adult), cultured normal and immortalized keratinocytes, and melanoma cells	T cell, melanoma cell	Inhibited by novel hydroxy derivatives of vitamin D	(5)

RORγ	Melanoma	Melanoma tissues		Decreased Positive associated with melanoma progression and shorter disease-free and overall survival	(24)

RORγ	Melanoma	Benign (nevi) and malignant (melanomas) melanocytic tumors	Keratinocytes, melanoma cells	Decreased Higher nuclear levels of RORγ and of cytoplasmic RORγ correlated with significantly longer overall and disease-free survival time	(68)

RORγ	Lung cancer	NSCLC tissues	Lung cancer cell	Increased High RORC2 expression leads to worse overall survival	(4)

RORγ	Lung cancer	Peripheral blood of NSCLC patients	PBMCs	Decreased	(69)

RORγ	Lung cancer	Peripheral blood of NSCLC patients	PBMCs	Increased Positively correlated with Th17 but negatively correlated with FOXP3	(70)

RORγ	Lung cancer	Peripheral blood of NSCLC patients	PBMCs	Increased Positively correlated with Th17 but negatively correlated with IL-27	(71)

RORγ	Lung cancer	Peripheral blood of NSCLC patient	PBMCs	Increased FoxP3/RORγ is higher in stage IV NSCLC patients than those of patients in stages I, II, and III	(72)

RORγ	Lung cancer	ADC and SSC tissues	Unspecified	Higher in the tumoral region of ADC compared with squamous cell carcinoma	(73)

RORγ	Hepatoma	Peripheral blood of hepatoma patients	PBMCs	Increased	(74)

RORγ	Hepatoma	Patients of steatosis/steatohepatitis, liver fibrosis, and HCC		Decreased	(27)

RORγ	Gastric cancer	Human gastritis and gastric ADC tissues, gp130^F/F^ mice that spontaneously develop gastric inflammation-associated tumors		Increased Not correlated with gastric tumorigenesis	(75)

RORγ	Colorectal cancer	Human CRC tissues	Foxp3^+^IL-17^+^ cells	Increased	(76)

RORγ	Colorectal cancer	Tissues and peripheral blood of colorectal cancer patients and RORγt-deficient mice	RORγ^+^ Treg cells	Increased Deficiency in RORγt protects against polyposis and improve cancer immune surveillance	(77)

RORγ	Colorectal cancer	Itch^−/−^ mice	Th17 cells; innate lymphoid cells	Regulated by itch Inhibition or genetic inactivation of RORγ attenuated IL-17 expression and reduced spontaneous colonic inflammation in Itch^−/−^ mice	(78)

RORγ	Colorectal cancer	Human CRC tissues	Unspecified	Unchanged	(79)

RORγ	Prostate cancer	Primary prostate cancer and metastatic prostate cancer samples	Prostate cancer cell	Increased Overexpressed and amplified in metastatic CRPC tumors Directly controls AR gene expression	(35)

RORγ	Cervical cancer	Peripheral blood of patients with cervical cancer or CIN	PBMCs	Increased Positively correlation with Th17 cells and Th22 cells in CIN and cervical cancer patients	(80)

RORγ1	Fibrosarcoma	BM or spleens from fibrosarcoma mice model Patients with T2 or T3 CRC	Myeloid cells	Drives cancer-related myelopoiesis in response to colony-stimulating factors Suppresses negative (Socs3 and Bcl3) and promotes positive (C/EBPb) regulators of granulopoiesis Promotes the protumor differentiation of MDSCs and TAMs	(65)

*ER-positive, estrogen receptor positive; BC, breast cancer; SSCC, skin squamous cell carcinoma; IL-17^Hi^, high expression of IL-17; IDC, invasive ductal carcinoma of the breast; LN, tumor-draining lymph nodes; ILC3, group 3 innate lymphoid cells; NSCLC, non-small cell lung cancer; DMFS, distance metastasis-free survival; PBMC, peripheral blood monocyte cell; ADC, adenocarcinoma; SCC, squamous cell carcinoma; HCC, hepatocellular carcinoma; CRC, colorectal cancer; CC, colon cancer; CIN, cervical intraepithelial neoplasia; CRPC, castration-resistant prostate cancer; AR, androgen receptor; ROREs, ROR-responsive elements; RORs, receptor-related orphan receptors; EMT, epithelial–mesenchymal transition; RFS, relapse free survival; Treg, regulatory T cell; CCICs, colorectal cancer-initiating cells*.

The natural expression of RORβ is exclusively restricted to neuronal tissues; therefore, activation of RORβ transcription is predominantly found in neuroblastoma cell lines (56), and literature on the role of RORβ in cancer is not much. Nevertheless, primary uterine leiomyosarcoma showed high RORβ expression (30), pharyngeal carcinoma cells and colorectal cancer cells showed modulated RORβ expression (29, 31), and RORβ was related to metastasis in a metastatic colorectal cancer cell model (28), which are summarized in Table 1. Based on the studies mentioned above, RORβ shows features of a tumor suppressor (Figure 1), but the potential roles of RORβ in various cancers related processes such as tumor proliferation and metastasis warrant further investigation.

### RORγ in Various Cancers

On the contrary, RORγ and its isoforms are extensively found in various kinds of malignancies. The diverse roles of RORγ in distinct cancers are specifically described below and summarized in Table 1 and Figure 1.

#### Hematological Malignancies

RORγ was found to function as an important element in lymphatic tumors (32), and mice deficient in RORγ were shown to have a high incidence of lymphoma metastasis and death within 4 months (57). Moreover, RORγ is frequently studied in tumor-infiltrating immune cells. RORγ mRNA expression in total lymphocytes was found unchanged between multiple myeloma and healthy controls (58, 59), but it was identified upregulation in peripheral blood monocyte cell (PBMC) from multiple myeloma comparing with healthy controls (60).

#### Breast Cancer

RORγ was found to be significantly overexpressed among infiltrating IL-17^+^ T cells, which drive immunosuppression in BC (61), and in breast tumor tissues compared with control tissues (62). An investigation related to group 3 innate lymphoid cells (ILC3) in BC revealed a role for RORγt + ILC3 in promoting lymph node metastasis by modulating chemokines in the tumor microenvironment (63). RORγ was found to be decreased in basal-like and grade 3 BCs, and inhibition of RORγ blocked cell viability, migration, and epithelial–mesenchymal transition (EMT) (64). However, an earlier study suggested that high expression of RORγ1, but not RORγt, by cancer cells was related to a high distance metastasis-free survival and was inversely correlated with decreased expression of PRMT2, which could suppress cell migration in BC (33). Accordingly, the different functions of RORγ in BC may be due to distinct cell origins and isotypes. For instance, when expressed by immune cells, RORγ acts as an immune suppressor, although when produced by cancer cells, it acts as a potential survival factor.

#### Skin Cancer

RORγ1 regulated tumor-promoting “emergency” granulo-monocytopoiesis by suppressing negative (Socs3 and Bcl3) and promoting positive (C/EBPb) regulators of granulopoiesis and RORγ1 promoted expansion of tumor-promoting MDSCs and TAM in fibrocarcinoma mice models (65). In a study exploring the function of Th17 cells in antitumor immunity, RORγt was found to be expressed by tumor-infiltrating Th17 cells. Th17 cells did not exhibit *in vitro* tumor cell killing activity, although CD8^+^ cytotoxic T cells stimulated by Th17 cells could activate the tumor killing response in a mouse B16 melanoma model (66).

In another study, RORγ-deficient mice showed inhibited melanoma growth, and this effect was identified to be IL-9 dependent (67). Together with RORα, RORγ was found to be expressed in melanoma cell lines and could bind with vitamin D3 derivatives, including 20(OH)D3 and 20,23(OH)2D3 (5), active forms of secosteroids and lumisterol can have anti-melanoma activity through action on RORα and RORγ (22, 24, 25, 39). In another study, RORγ and RORα expression levels were decreased during melanoma progression, with the lowest expression levels in stages III and IV primary melanomas and in melanoma (68). These studies of RORα and RORγ in melanoma suggest that RORα and RORγ could be important modulators affecting melanomagenesis, contributing to the anti-melanoma activity of vitamin D3 and act as potential therapeutic targets in adjuvant melanoma therapy (23, 24). The investigation of RORγ in skin cancer seems to be concentrated on melanoma and the isotype RORγ1, thus, there is a need for further exploration focusing on the regulation of RORγ and its roles in other types of skin cancer.

#### Lung Cancer

Our previous study showed that RORγ2 was highly expressed in non-small cell lung cancer (NSCLC) cells and also served as a prognostic factor (4). The expression of RORγt mRNA and protein was found to be downregulated in PBMCs from NSCLC patients compared with controls (69). However, RORγt mRNA was found to be upregulated in the peripheral blood of patients with NSCLC compared with that of healthy controls (70), which was confirmed in other studies (71, 72). Moreover, in a recent report, RORγt, together with Th17/IL-6R/pSTAT3/BATF, was upregulated in the tumor region of adenocarcinomas, except for squamous carcinomas of lung cancer (73). Studies focused on cancer cell-derived RORγt are infrequent and require additional attention.

#### Hepatocellular Carcinoma

RORγt mRNA was shown to be increased in HCC compared with a normal control group (74). By contrast, RORγt mRNA expression was found to be significantly lower in patients with steatosis/steatohepatitis, liver fibrosis, and HCC (27). Investigations into RORγt in HCC are rare, although RORγt is known to be expressed in hepatocytes. There could be additional modulatory roles for RORγt in HCC progression, and further studies are warranted.

#### Gastrointestinal Cancer

The gene expression of IL-17A and RORγ was not altered in gastric cancer (75). Foxp3^+^IL-17^+^ cells in colorectal cancer were found to express RORγt (76). Another study described RORγt-expressing regulatory T cells that were linked with the inability of these cells to suppress inflammation and were directly associated with the stage of human colon cancer (77). RORγt was also found to be involved in inhibiting colon carcinogenesis through binding with an E3 ubiquitin ligase, Itch, for ubiquitination (78). However, RORγt was not expressed within colorectal cancer tissues or by colorectal cancer-infiltrating CD4^+^ T cells (79). The expression and regulation of RORγt in gastric and colorectal cancer remain controversial, which makes it difficult to conclude the extent of RORγ/RORγt expression or the involvement in tumorigenesis. However, the differences in results from different studies might be attributable to the diversity of detection methods from tissue samples when considering individual variation.

#### Genitourinary Cancer

In castration-resistant prostate cancer (CRPC), RORγ was examined as a therapeutic target due to its overexpression and was found to directly drive androgen receptor (AR) hyperactivity through binding to an exonic RORE and partly through the NR coactivators SRC-1 and -3 (35). Therefore, inhibition of RORγ may represent a possible treatment option for CRPC. The transcriptional expression of RORγ mRNA from PBMCs exhibited high levels in cervical cancer compared with healthy controls (80). Additional observations are needed to elucidate the functions of RORγ in genitourinary cancer, where it may serve as a valuable therapeutic target.

## Perspective

The three ROR family members are regarded as important regulators of the circadian rhythm, metabolism, and tumorigenesis. As discussed in this review, the protumor or antitumor effects of RORα and RORβ in cancer have not been intensively explored, requiring further study and evidence. However, as the main transcription factor in IL-17-expressing immune cells, RORγ has been investigated in various cancer cells and tumor-infiltrating cells (Figure 1), indicating that it might be a promising prognostic factor in lung and BC and a potential therapeutic target in prostate cancer.

Moreover, according to this review, we could conclude that the roles that RORs family members play in tumorigenesis vary in different cancers and, to some extent, depend on producing cells in the tumor microenvironment. Further concentration on the relationships between RORs and tumorigenesis should be meticulously organized and should deeply explore the clinical significance and the underlying mechanisms. More importantly, each RORs family members consists of several isoforms, and some previous studies have showed that different RORs isoforms present different biological functions (6). Thus, prospective reports on therapeutic targets of RORs in cancer should identify all isoforms of specific RORs.

Since RORα and RORγ are dysregulated in multiple cancer types based on published articles, they likely participate in carcinogenesis through modulating molecules such as IL-17, PRMT2, and AR or as receptors for sterols, such as vitamin D3 derivatives. Intriguingly, IL-17, AR, and vitamin D3 are therapeutic targets in rheumatoid arthritis and have potential, as a frontline treatment option for advanced prostate cancer and an adjuvant in melanoma management. Agonists or inverse agonists for RORα and RORγ might be efficiently inhibiting tumor growth and progression through activation or inactivation so that their ligands or targets, such as vitamin D3 derivatives and AR, become valid or invalid. Another promising new strategy for anticancer therapy might involve directly targeting tumor cells with RORα- and RORγ-specific modulators due to the correlations between high or low expression of RORα and RORγ and tumor progression. Third, RORs are sometimes produced by immune cells in tumor microenvironments and then induce antitumor or protumor activity by regulating tumor-related cytokines or chemokines. Accordingly, therapies targeting RORs producing immune cells could be novel treatments for certain cancers.

## Author Contributions

JF, ZL and GY wrote the draft. YJ revised the manuscript. JF, TL, JX, and FW designed the figures. QH, MG, GH, MZ, LD and SL commented and added extra information.

## Conflict of Interest Statement

The authors declare that the research was conducted in the absence of any commercial or financial relationships that could be construed as a potential conflict of interest.
